# Association between Sick Leave Prescribing Practices and Physician Burnout and Empathy

**DOI:** 10.1371/journal.pone.0133379

**Published:** 2015-07-21

**Authors:** Oriol Yuguero Torres, Montserrat Esquerda Aresté, Josep Ramon Marsal Mora, Jorge Soler-González

**Affiliations:** 1 Primary Care Service, Lleida Health Region, Hospital Arnau de Vilanova, Lleida, Spain; 2 Borja Bioethics Institute, Esplugues, Barcelona, Spain; 3 School of Medicine University of Lleida, Lleida, Spain; 4 Jordi Gol Institute for Research in Primary Care IDIAP Jordi Gol, Lleida, Spain; University of Geneva, SWITZERLAND

## Abstract

**Objectives:**

To investigate the association between sick leave prescription and physician burnout and empathy in a primary care health district in Lleida, Spain.

**Methods:**

This descriptive study included 108 primary care doctors from 22 primary care centers in Lleida in 2014 (183,600 patients). Burnout was measured with the Maslach Burnout Inventory and empathy with the Jefferson Scale of Physician Empathy. The reliability of the instruments was measured by calculating Cronbach’s alpha and normal distribution was analyzed using the Kolmogorov-Smirnov-Lilliefors and χ^2^ tests. Burnout and empathy scores were analyzed by age, sex, and place of work (urban vs rural). Sick leave data were obtained from the Catalan Health Institute.

**Results:**

High empathy was significantly associated with low burnout. Neither empathy nor burnout were significantly associated with sick leave prescription.

**Conclusion:**

Sick leave prescription by physicians is not associated with physicians' empathy or burnout and may mostly depend on prescribing guidelines.

## Introduction

Sick leave is a medical situation in which a patient is granted leave of absence due to a non-work-related illness or injury [1]. In Spain, it is evaluated by primary care physicians and patients granted leave qualify for sick pay from the state.

The physician-patient relationship is a fundamental element of clinical practice that has changed throughout the history of medicine [2]^,^[3]. The roles of both physicians and patients have evolved hand in hand with scientific developments and social changes, particularly in the twentieth century. The most profound changes, however, have probably come about in recent years, in part due to the increasing use of computers and technology.

A recent review reflecting on why some physicians become “unethical or even callous,” identified multiple causes, including difficulties related to emotional and relational aspects, such as emotional overload from contact with suffering, the limitations of medicine in terms of resolving certain patient problems, and the contradiction between the ideals of the profession and its day-to-day reality, characterized by high demands from patients and overburdened physicians [4].

The term “burnout” first appeared in the medical literature in 1974 [5]; it was coined by Freudenberger to describe wear and/or professional overload in a group of workers. However, Maslach’s work [6] on the symptoms of burnout among professionals, and particularly those working with the public, led to the widespread use of the term [7]. In 1986, Maslach’s work culminated in the creation of a 22-item scale designed to quantify this new syndrome [8], which according to Maslach consisted of three main characteristics [9]: fatigue or emotional exhaustion, depersonalization and dehumanization, and a low sense of personal accomplishment.

Burnout symptoms are similar to those seen in situations of chronic stress and can be grouped into [10]^,^[11] psychosomatic symptoms (e.g., headache, fatigue, gastrointestinal disorders) and behavioral, emotional, and/or defensive symptoms.

Low empathy seems to be related to high levels of burnout and in particular to certain components of burnout, such as emotional exhaustion, depersonalization, and a low sense of personal accomplishment [12].

Physician empathy refers not only to a physician’s attitude towards and ability to understand the experiences and feelings of their patients, but also to their capacity to communicate these feelings. However, empathy has also been associated—both theoretically and empirically—with a number of attributes such as respect, prosocial behavior, moral reasoning, positive attitudes towards elderly people, the ability to create a good medical history, physician and patient satisfaction, and good clinical outcomes [13].

Empathy is an essential component of the physician-patient relationship and is probably related to clinical benefits for the patient. Although an empathic relationship suggests the existence of a good physician-patient relationship, there are few objective data to support this[14]^,^[15].

Several studies have provided evidence that a good physician-patient relationship has a positive influence on clinical results and patient outcomes[16]^,^[17]. Indeed, educational and professional organizations recommend that empathy be recognized as a fundamental quality of a good medical professional[18].

Some studies suggest that indicators of empathic involvement in patient care (such as good verbal or nonverbal communication with the patient) and duration of visits may lead to increased patient satisfaction [19]^,^[20]^,^[21]^,^[22] and greater treatment adherence [23]^,^[24]^,^[25]. Empathy has been linked to psychotherapeutic efficacy [26]^,^[27], patients’ sense of being important, and diagnostic and prognostic accuracy [28]^,^[29], and has also been described as the most important quality of a “good doctor” [30]. However, few studies have found an association between empathy and clinical results (e.g. laboratory results) as an intermediate step towards a better overall outcome. A 2011 study of patients with diabetes showed a positive association between physician empathy and clinical outcomes and reported that an understanding of the patient’s perspective (something fundamental to empathy) both reinforced the patient’s perception of being helped and increased his or her perception of social support [31]. The authors confirmed that physician empathy is linked to clinical benefits for the patient and suggested that empathy should be considered when assessing physician competence. Degree of empathy may also be influenced by a physician’s specialty, as professionals who interact more with people have been observed to score higher in empathy tests than those who engage in surgery or more technical specialties[28].

Few studies have investigated the association between physician burnout and empathy, even though, as claimed by Brazeau et al [32] there appears to be an evident association between the two.

The objectives of this study were to evaluate the level of burnout and empathy in primary care physicians in the Catalan health care district of Lleida (Spain) and to investigate associations with sick leave prescribing practices.

## Methodology

### Participants

We e-mailed all 217 general practitioners (GPs) working in the health care district of Lleida, a province in Catalonia with a population of approximately 300.000, asking them to participate in the study, which consisted of completing 2 online instruments measuring empathy and burnout. In total, 133 GPs (61.2%) agreed to participate and provided written informed consent. All items in both instruments were completed correctly by 108 participants (81.2%), who were included in the final data analysis. These 108 GPs attended 183,600 patients and were attached to 22 primary health care centers. The survey was conducted between May and July 2014.

### Instruments

Burnout was measured using the Spanish version of the Maslach Burnout Inventory (MBI), validated by Moreno-Jimenez. [33] The MBI is a scale composed of 22 items, which are scored on a 7-point Likert-type frequency scale ranging from 0 (never) to 6 (every day). This scale is widely recognized and has been used in a multitude of projects [34] and studies by both physicians [35] and nurses [36].

Empathy was measured using the Jefferson Scale of Physician Empathy (JSPE) [37]. The JSPE is a 20-item 7-point scale Likert-type scale scored from 1 (strongly disagree) to 7 strongly agree). We also used a validated Spanish version of the JSPE[38].

### Other variable

The health care centers were classified as urban (those in the city of Lleida, the capital of the region) or rural (those in other towns and villages). The following data were collected for each GP: age, sex, place of work (urban or rural).

Sick leave data were obtained from the Catalan Health Institute’s primary health care database, which contains records for all patients seen under the Catalan primary health care system. The number of Sick Leave and its duration was calculated by each patient. One of the authors (JM), a biostatistician, had access to this database.

### Study design and data analysis

A descriptive analysis of the qualitative variables and burnout and empathy scores was performed. The reliability of the MBI and JSPE was measured by calculating Cronbach’s alpha, and normal distribution of data was tested using the Kolmogorov-Smirnov-Lilliefors and χ^2^ tests. The Pearson correlation coefficient was used for normally distributed data and Spearman’s rank correlation coefficient was used for non-normally distributed data.

In cases of asymmetric distribution quotas related to the age characteristics of patients, data were fitted to a linear regression model. We analyzed the association between the different sociodemographic variables analyzed and the results for the JSPE and MBI tests. The GPs were divided into three groups according to their level of empathy and burnout (low, moderate, and high in each case).

Results were also analyzed by age, sex, and place of work (urban vs rural).

For the data analysis, descriptive means of frequency, percentages, and standard deviations were calculated using SPSS version 15.0.

### Ethical and confidentiality aspects

The study, survey, and written consent procedure were approved by the clinical research ethics committee of the Jordi Gol Institute for Research in Primary Care (IDIAP). Confidentiality and anonymity were ensured under the Spanish Data Protection Law. All data were coded and accessible only to JM, who cross-referenced the health care and participant data. Because the database was anonymous, at no time were the researchers able to identify the study participants.

## Results

Table 1 summarizes the sociodemographic characteristics of our sample according to level of empathy (low, moderate, high) and also shows the relationship between level of burnout and level of empathy. In the high empathy group there was a predominance of female doctors (72.1%). The most remarkable finding to emerge was that high empathy was significantly associated with low professional burnout (p<0.05). No significant associations were found between empathy and sex, age, or work place (urban vs rural).

**Table 1:** Association between empathy and sociodemographic variables and burnout.

	Empathy			
	Low (n = 33)	Moderate (n = 32)	High (n = 43)	p
Sex (female)	21 (63.6%)	17 (53.1%)	31 (72.1%)	0.388
Age (years)	50.7 (9.34)	49.1 (8.07)	48.3 (8.06)	0.447
Working in rural area	24 (72.7%)	14 (43.8%)	21 (48.8%)	0.051
Burnout				0.001
Low	12 (36.4%)	17 (53.1%)	31 (72.1%)	
Moderate	17 (51.5%)	13 (40.6%)	11 (25.6%)	
High	4 (12.1%)	2 (6.3%)	1 (2.3%)	
Emotional Exhaustion				0.135
Low	17 (51.5%)	16 (50%)	26 (60.5%)	
Moderate	2 (6.1%)	7 (21.9%)	8 (18.6%)	
High	14 (42.4%)	9 (28.1%)	9 (20.9%)	
Depersonalization				0.006
Low	13 (39.4%)	19 (59.4%)	33 (76.7%)	
Moderate	10 (30.3%)	9 (28.1%)	3 (7%)	
High	10 (30.3%)	4 (12.5%)	7 (16.3%)	
Personal Accomplishment				<0.001
Low	8 (24.2%)	2 (6.3%)	2 (4.7%)	
Moderate	18 (54.5%)	15 (46.9%)	9 (20.9%)	
High	7 (21.2%)	15 (46.9%)	32 (74.4%)	

We studied all the components of burnout, but the only significant result was observed for personal accomplishment. In this case, we found that the most empathic GPs are those who feel a sense of competence and achievement in their work (p<0.05).

Table 2 summarizes our results in relation to sick leave prescription, with no significant associations observed for either empathy or burnout. Physicians with high burnout had a slightly higher percentage of patients on sick leave than those with low burnout (6.5% vs 6.2%of GPs), and also prescribed longer leave (mean 25.3 vs 28 days), but the differences were not enough to be considered statistically significant.

**Table 2:** Sick leave prescription according to empathy and burnout.

	**Burnout**						
	**Low (n = 60)**		**Moderate (n = 41)**		**High (n = 7)**		**p**
	**N**	**mean (SD)**	**N**	**mean (SD)**	**N**	**mean (SD)**	
Percentage of patients on sick leave	60	6.2 (1.6)	41	6.7 (1.8)	7	6.5 (1.4)	0.243
Duration of sick leave (days)	60	25.3 (5.9)	40	26.2 (5.3)	7	28 (5.7)	0.453
Sick leave granted to patients already granted sick leave in previous year (no.)	60	1.2 (0.1)	40	1.2 (0.1)	7	1.2 (0.1)	0.954
	**Empathy**						
	**Low (n = 33)**		**Moderate (n = 32)**		**High (n = 43)**		**P**
	**N**	**mean (SD)**	**N**	**mean (SD)**	**N**	**mean (SD)**	
Percentage of patients on sick leave	33	6.5 (1.5)	32	5.9 (1.9)	43	6.7 (1.6)	0.263
Duration of SL (days)	33	25 (4.7)	31	27.7 (7.2)	43	25.1 (4.8)	0.119
Sick leave granted to patients already granted sick leave in previous year (no.)	33	1.2 (0.1)	31	1.2 (0.1)	43	1.2 (0.1)	0.438

**Fig 1:**
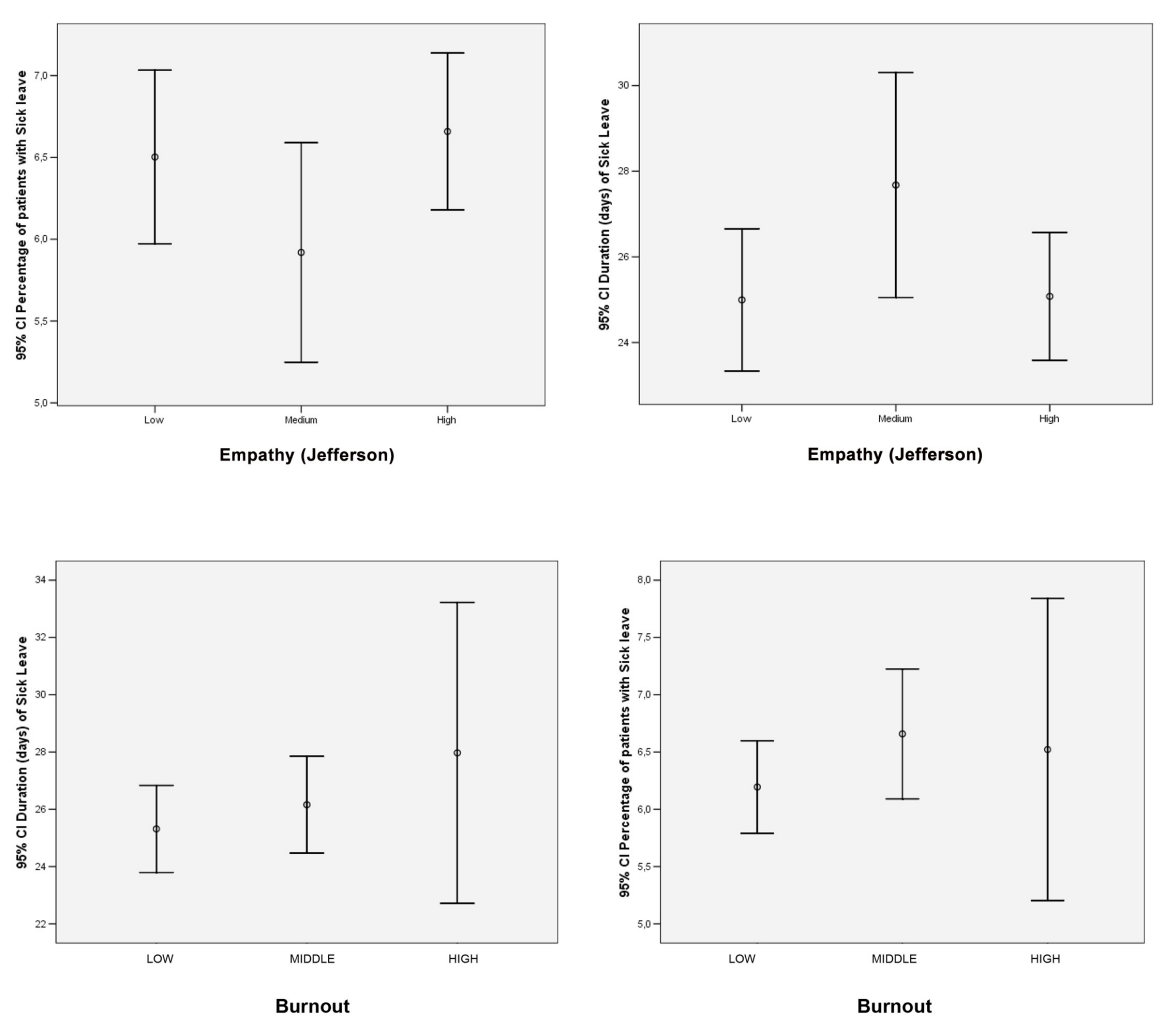
Association between burnout and empathy and sick leave prescription and duration.

The associations between sick leave prescription and both empathy and burnout are shown in Fig 1. While low burnout was associated with a lower rate of prescription and both high and low empathy were associated with a higher rate of prescription, none of these results were statistically significant (p>0.05).

## Discussion

To our knowledge, this is the first study to investigate the association between physician burnout and empathy and sick leave prescribing practices. One Spanish article analyzed the factors determining patient sick leave[39] but did not investigate physician-related factors, and while more recent studies have analyzed the association between sick leave prescribing habits and professional factors, such as physician sex[40] and place of practice[41], they did not investigate burnout or empathy. The present study is the first in an ongoing project evaluating empathy and professional burnout in primary health care professionals.

Although we found a non-significant association between sick leave prescription and either empathy and burnout we can not conclude absence of association. Note that it can be assumed a low trend between the percentage and the duration. Our study had a global low sample size for some groups (i.e. high burnout group there are only 7 professionals) that implies a low statistical power to detect small effects.

We did, however, find a significant association between higher empathy and lower burnout, and this association was particularly strong for certain aspects of burnout, like depersonalization (p<0.05). Promoting empathy among health professionals is a measure to reduce professional burnout. Professionals with higher empathy have a closer relationship with their patients, and approach their job with more energy and enthusiasm, possibly explaining why the GPs in our series reported greater job fulfilment (p<0.05). Satisifed GPs tend to respond better to patient’s demands [7]. Our working hypothesis was that more empathic doctors would understand their patients’ problems better and be more prone to granting sick leave, but this could not be proved because either really no association exists or because there was a low statistical power.

As our results suggest, sick leave prescribing practices probably depend on various factors but not empathy or burnout. In our series, GPs with high and low levels of empathy had similar sick leave prescribing practices, while those with more moderate levels were more likely to grant leave of absence. These results suggest that physicians in our region take a professional approach to their job and are not influenced by patient demands.

The strengths of our study include a relatively high response rate from a group of primary care physicians attending a large population of patients (n = 183,600) from an entire health district and the use of validated tests to measure both empathy and burnout. Finally, as already mentioned, to our knowledge this is the first study to investigate these relationships. This study can be considered as a pilot study what can be a first step for future researches.

Our study also has some limitations. Surveys on socially sensitive topics such as empathy hold numerous challenges, including the elicitation of truthful answers. To minimize this problem, we used the widely accepted and validated JSPE. It should be emphasized that the demographic characteristics of the health care centers analyzed are not known because the data analyzed were anonymized to enable a comprehensive analysis. Furthermore, because of a lack of previous similar studies, we are unable to compare our findings with those from other settings. We have only analyzed primary care professionals and it would be interesting to analyze a greater range of health professionals in future studies. It would also be interesting to compare our findings with data from other regions in Spain and to compare results between Spanish physicians and foreign physicians working in Spain (all the GPs in our series were Spanish) to evaluate if there are cultural factors affecting empathy. Finally, future studies should assess other factors that may explain burnout and empathy (e.g. personal, family, social, or work environment factors). Finally, of the 133 GPs who completed the survey, we were only able to analyze the sick leave prescribing practices of 108 of these due to incomplete data.

The most remarkable finding of the present study is that sick leave prescribing practices in our primary health care district are either not influenced by physician burnout or empathy, or if really exists association this must be low. However, further research with larger samples is needed to confirm our results.

In summary, our observation that burnout and empathy have not a high influence sick leave prescribing practices among primary care physicians have implications for the future, as these practices obviously depend on other factors. This has implications for the design of interventions aimed at improving the professional practice of health professionals. Research has shown that professional low burnout and empathy with patients are associated with positive personality attributes that lead to a healthy clinical relationship[42],[43], and it is probably good for patients that sick leave prescription depends on objective rather than subjective or behavioral factors. Considering that sick leave prescription has important socioeconomic repercussions, and particularly in the current climate, it is reassuring to see that primary care physicians in our health care district appear to be guided by professional judgment and are not influenced by burnout or empathy.

## Supporting Information

S1 Table Variable Definition of the Study. PDF File with all the variable definition.
(PDF)

S2 Table Table with all data of professionals of the study. (PDF)
