# Supplementary material for: Association between Sick Leave Prescribing Practices and Physician Burnout and Empathy
Source: PLoS One. 2015 Jul 21;10(7):e0133379. doi: 10.1371/journal.pone.0133379 (PMC4510532; doi:10.1371/journal.pone.0133379)
Supplement: S1 Table — PDF File with all the variable definition. (PDF) [file pone.0133379.s001.pdf]

Order	Variable Name	Definition	Type	Levels
1	mbic	*Burnout*	*Categorical (3)*	1: Low / 2: Moderate / 3:High
2	b_ee	*Emotional Exhaustion*	*Categorical (3)*	1: Low / 2: Moderate / 3:High
3	b_d	*Despersonalization*	*Categorical (3)*	1: Low / 2: Moderate / 3:High
4	b_pa	*Personal Accomplishment*	*Categorical (3)*	1: Low / 2: Moderate / 3:High
5	totalc	*Empathy*	*Categorical (3)*	1: Low / 2: Moderate / 3:High
6	sex	*Sex*	*Categorical (2)*	0: Male / 1:Female
7	age	*Age (years)*	*Numerical*	
8	sl	*Percentage of patients on sick leave*	*Numerical*	
9	duration	*Duration of Sick Leave (days)*	*Numerical*	
10	num	*Sick leave granted to patients already granted sick leave in previous year*	*Numerical*	
11	id	*Professional Identification*	*Numerical*	
