# Supplementary material for: Association between Sick Leave Prescribing Practices and Physician Burnout and Empathy
Source: PLoS One. 2015 Jul 21;10(7):e0133379. doi: 10.1371/journal.pone.0133379 (PMC4510532; doi:10.1371/journal.pone.0133379)
Supplement: S2 Table — (PDF) [file pone.0133379.s002.pdf]

mbic	b_ee	b_d	b_pa	totalc	sex	age	sl	duration	num	id
1,00	1,00	1,00	3,00	3,00	1,00	41,00	7,29	20,75	1,16	1,00
2,00	3,00	3,00	2,00	1,00	1,00	60,00	7,88	24,40	1,26	2,00
2,00	1,00	3,00	1,00	1,00	0,00	58,00	4,58	34,66	1,10	3,00
1,00	1,00	1,00	3,00	1,00	1,00	43,00	8,73	21,62	1,26	4,00
2,00	3,00	3,00	2,00	3,00	0,00	61,00	6,52	36,23	1,14	5,00
3,00	3,00	3,00	2,00	2,00	1,00	48,00	6,35	36,74	1,25	6,00
1,00	1,00	1,00	2,00	2,00	0,00	48,00	5,57	24,31	1,17	7,00
1,00	1,00	1,00	3,00	3,00	1,00	39,00	4,97	26,57	1,16	8,00
1,00	1,00	1,00	3,00	3,00	1,00	47,00	6,92	28,71	1,27	9,00
2,00	2,00	2,00	3,00	3,00	1,00	54,00	6,83	29,11	1,33	10,00
1,00	1,00	2,00	2,00	1,00	1,00	61,00	5,27	22,16	1,27	11,00
1,00	1,00	1,00	2,00	2,00	0,00	55,00	6,48	27,04	1,36	12,00
1,00	1,00	1,00	2,00	1,00	1,00	57,00	4,95	27,57	1,17	13,00
1,00	1,00	1,00	3,00	2,00	0,00	59,00	6,01	26,62	1,13	14,00
1,00	1,00	2,00	3,00	2,00	1,00	48,00	2,85	53,29	1,08	15,00
2,00	3,00	1,00	3,00	2,00	0,00	57,00	5,88	28,57	1,19	16,00
1,00	1,00	1,00	2,00	2,00	1,00	47,00	5,40	23,45	1,27	17,00
2,00	3,00	2,00	2,00	2,00	1,00	48,00	8,12	24,28	1,29	18,00
2,00	3,00	3,00	1,00	3,00	1,00	41,00	6,87	18,82	1,18	19,00
2,00	3,00	3,00	2,00	2,00	0,00	36,00	0,00	0,00	0,00	20,00
1,00	1,00	1,00	3,00	3,00	1,00	43,00	5,45	26,00	1,36	21,00
1,00	1,00	1,00	3,00	1,00	0,00	54,00	5,56	30,24	1,15	22,00
2,00	3,00	1,00	2,00	1,00	1,00	42,00	5,89	30,22	1,12	23,00
2,00	2,00	1,00	3,00	3,00	1,00	56,00	8,09	21,00	1,32	24,00
1,00	1,00	1,00	3,00	1,00	1,00	62,00	5,78	19,65	1,29	25,00
2,00	3,00	1,00	3,00	1,00	1,00	52,00	6,78	32,76	1,27	26,00
2,00	3,00	3,00	3,00	1,00	0,00	57,00	8,99	20,51	1,26	27,00
1,00	1,00	1,00	3,00	2,00	1,00	37,00	4,62	24,31	1,24	28,00
1,00	2,00	1,00	2,00	3,00	0,00	47,00	5,51	34,87	1,22	29,00
2,00	3,00	3,00	2,00	3,00	0,00	58,00	5,63	29,02	1,24	30,00
2,00	3,00	1,00	3,00	3,00	1,00	45,00	10,05	25,32	1,42	31,00
3,00	3,00	3,00	1,00	1,00	0,00	54,00	5,96	25,46	1,21	32,00
1,00	2,00	1,00	3,00	3,00	0,00	60,00	5,47	20,43	1,13	33,00
1,00	1,00	1,00	3,00	3,00	0,00	56,00	6,09	23,97	1,21	34,00
1,00	1,00	1,00	3,00	1,00	0,00	56,00	6,24	15,97	1,41	35,00

2,00	1,00	2,00	2,00	1,00	1,00	55,00	5,99	22,42	1,21	36,00
1,00	1,00	1,00	3,00	3,00	1,00	44,00	6,60	24,14	1,14	37,00
1,00	1,00	2,00	3,00	3,00	1,00	44,00	8,81	24,87	1,16	38,00
3,00	3,00	3,00	1,00	2,00	0,00	44,00	9,48	22,06	1,35	39,00
2,00	3,00	2,00	3,00	2,00	1,00	53,00	6,08	29,32	1,16	40,00
2,00	2,00	1,00	2,00	2,00	1,00	36,00	3,77	30,58	1,31	41,00
1,00	1,00	1,00	2,00	1,00	0,00	58,00	7,25	20,26	1,38	42,00
2,00	1,00	3,00	1,00	2,00	1,00	42,00	5,23	19,80	1,21	43,00
3,00	3,00	3,00	1,00	1,00	0,00	52,00	5,71	28,62	1,15	44,00
2,00	2,00	1,00	3,00	2,00	1,00	47,00	7,60	20,62	1,20	45,00
3,00	3,00	3,00	2,00	1,00	1,00	65,00	7,00	21,18	1,22	46,00
1,00	1,00	1,00	3,00	2,00	0,00	59,00	5,92	28,70	1,26	47,00
1,00	1,00	2,00	2,00	2,00	0,00	61,00	6,16	16,34	1,40	48,00
2,00	1,00	1,00	2,00	2,00	1,00	36,00	8,59	28,93	1,19	49,00
3,00	3,00	3,00	2,00	1,00	0,00	61,00	6,01	28,41	1,31	50,00
1,00	1,00	1,00	3,00	2,00	1,00	48,00	7,57	27,19	1,22	51,00
1,00	2,00	1,00	2,00	2,00	1,00	51,00	6,88	27,61	1,26	52,00
1,00	1,00	1,00	1,00	1,00	1,00	43,00	2,59	33,71	1,42	53,00
1,00	1,00	1,00	3,00	3,00	1,00	40,00	4,67	34,79	1,12	54,00
1,00	1,00	1,00	3,00	3,00	1,00	33,00	5,90	21,01	1,15	55,00
1,00	1,00	1,00	3,00	3,00	0,00	62,00	6,96	24,88	1,11	56,00
2,00	2,00	1,00	1,00	1,00	0,00	60,00	7,94	23,67	1,17	57,00
2,00	3,00	1,00	2,00	3,00	1,00	41,00	8,12	26,09	1,25	58,00
1,00	1,00	1,00	3,00	3,00	1,00	45,00	6,41	25,73	1,18	59,00
2,00	2,00	2,00	2,00	1,00	1,00	47,00	7,48	24,64	1,21	60,00
2,00	1,00	3,00	2,00	3,00	0,00	49,00	9,55	25,47	1,39	61,00
1,00	1,00	1,00	3,00	3,00	1,00	36,00	5,42	19,67	1,39	62,00
1,00	1,00	1,00	3,00	3,00	0,00	43,00	6,50	26,21	1,19	63,00
1,00	1,00	1,00	3,00	3,00	1,00	42,00	8,83	24,55	1,26	64,00
1,00	1,00	1,00	3,00	2,00	1,00	43,00	7,01	20,65	1,31	65,00
2,00	3,00	3,00	3,00	3,00	1,00	58,00	7,17	20,81	1,30	66,00
1,00	1,00	2,00	2,00	2,00	0,00	58,00	3,37	25,56	1,04	67,00
3,00	3,00	3,00	1,00	3,00	1,00	52,00	5,14	33,33	1,12	68,00
1,00	1,00	2,00	2,00	1,00	1,00	49,00	7,91	19,10	1,23	69,00
1,00	1,00	1,00	2,00	3,00	0,00	57,00	9,21	21,69	1,22	70,00
2,00	3,00	2,00	2,00	2,00	0,00	57,00	7,85	32,29	1,21	71,00

1,00	1,00	1,00	3,00	3,00	1,00	43,00	5,74	28,76	1,25	72,00
1,00	2,00	1,00	3,00	2,00	0,00	47,00	8,26	24,51	1,20	73,00
2,00	3,00	2,00	1,00	1,00	1,00	41,00	6,85	28,06	1,26	74,00
2,00	2,00	1,00	3,00	2,00	0,00	62,00	4,97	39,61	1,00	75,00
2,00	3,00	3,00	3,00	3,00	1,00	55,00	4,96	18,24	1,20	76,00
1,00	1,00	1,00	3,00	3,00	1,00	42,00	5,35	25,70	1,19	77,00
2,00	1,00	2,00	1,00	1,00	1,00	36,00	6,15	19,61	1,26	78,00
2,00	1,00	3,00	2,00	1,00	1,00	36,00	6,87	25,45	1,20	79,00
1,00	1,00	1,00	2,00	1,00	0,00	48,00	8,07	27,22	1,29	80,00
2,00	1,00	2,00	1,00	1,00	0,00	63,00	6,97	21,03	1,44	81,00
1,00	1,00	1,00	3,00	3,00	1,00	41,00	9,18	26,34	1,24	82,00
2,00	3,00	3,00	3,00	1,00	1,00	35,00	4,64	24,21	1,03	83,00
2,00	2,00	1,00	2,00	2,00	0,00	55,00	6,51	29,29	1,29	84,00
1,00	1,00	1,00	3,00	2,00	0,00	60,00	4,88	28,73	1,26	85,00
2,00	3,00	3,00	2,00	1,00	1,00	48,00	6,71	24,49	1,25	86,00
1,00	2,00	1,00	3,00	3,00	1,00	41,00	5,05	22,53	1,22	87,00
2,00	3,00	2,00	3,00	2,00	0,00	59,00	5,41	38,14	1,11	88,00
1,00	2,00	1,00	3,00	3,00	1,00	39,00	7,28	23,32	1,28	89,00
2,00	3,00	2,00	2,00	1,00	0,00	61,00	8,41	21,78	1,22	90,00
2,00	1,00	2,00	2,00	3,00	0,00	57,00	7,71	23,47	1,27	91,00
1,00	1,00	1,00	3,00	3,00	1,00	44,00	6,14	22,52	1,28	92,00
1,00	1,00	1,00	2,00	3,00	1,00	48,00	6,40	12,35	1,27	93,00
1,00	1,00	1,00	3,00	3,00	0,00	60,00	7,22	26,61	1,15	94,00
1,00	1,00	1,00	3,00	3,00	1,00	55,00	4,87	34,50	1,15	95,00
1,00	2,00	1,00	3,00	3,00	0,00	39,00	5,35	22,98	1,25	96,00
1,00	3,00	1,00	3,00	3,00	1,00	61,00	3,33	24,55	1,12	97,00
2,00	3,00	2,00	2,00	2,00	1,00	47,00	4,86	26,10	1,24	98,00
1,00	2,00	1,00	3,00	2,00	1,00	41,00	7,37	26,52	1,22	99,00
1,00	1,00	1,00	2,00	3,00	1,00	59,00	7,43	26,02	1,20	100,00
1,00	2,00	1,00	3,00	3,00	1,00	49,00	5,53	27,25	1,26	101,00
1,00	1,00	1,00	2,00	1,00	1,00	48,00	7,42	24,32	1,28	102,00
1,00	1,00	2,00	2,00	2,00	1,00	39,00	5,01	29,58	1,31	103,00
1,00	1,00	1,00	3,00	3,00	1,00	51,00	9,80	19,14	1,30	104,00
1,00	1,00	1,00	2,00	1,00	1,00	40,00	3,50	34,07	1,38	105,00
1,00	1,00	1,00	3,00	2,00	1,00	43,00	5,35	17,26	1,52	106,00
2,00	3,00	2,00	2,00	1,00	1,00	38,00	5,65	23,59	1,23	107,00

2,00	3,00	2,00	2,00	1,00	1,00	34,00	8,85	23,78	1,30	108,00
